# What are mass media interventions made of? Exploring the active content of interventions designed to increase HIV testing in gay men within a systematic review

**DOI:** 10.1111/bjhp.12377

**Published:** 2019-07-02

**Authors:** Paul Flowers, Julie Riddell, Nicola Boydell, Gemma Teal, Nicky Coia, Lisa McDaid

**Affiliations:** ^1^ MRC/CSO Social and Public Health Sciences Unit University of Glasgow UK; ^2^ Usher Institute of Population Health Sciences and Informatics University of Edinburgh UK; ^3^ Innovation School, The Glasgow School of Art UK; ^4^ NHS Greater Glasgow and Clyde UK

**Keywords:** behaviour change, behaviour change techniques, HIV testing, intervention content, men who have sex with men, review

## Abstract

**Purpose:**

Mass media HIV testing interventions are effective in increasing testing, but there has been no examination of their theory or behaviour change technique (BCT) content. Within a heterogeneous body of studies with weak evaluative designs and differing outcomes, we attempted to gain useful knowledge to shape future interventions.

**Methods:**

Within a systematic review, following repeated requests to the authors of included studies for intervention materials, the Theory Coding Scheme, the Theoretical Domains Framework (TDF), and Behaviour Change Technique Taxonomy (BCTT) were used to extract data relating to active intervention content.

**Results:**

Of 19 studies, five reported an explicit theoretical basis to their intervention. TDF analysis highlighted the key domains employed within the majority of interventions: ‘knowledge’, ‘social roles and identities’, and ‘beliefs about consequences’. BCT analysis showed three BCT groupings commonly reported within interventions: ‘Comparison of outcomes’, ‘Natural consequences’, and ‘Shaping knowledge’. Three individual BCTs formed the backbone of most interventions and can be considered ‘standard’ content: ‘Instructions on how to perform behaviour’; ‘Credible source’; and ‘Information about health consequences’.

**Conclusions:**

This is the first study to examine and detail active intervention content in this field. It suggests future interventions should improve knowledge about testing, and use well‐branded and trusted sources that endorse testing. Future interventions should also provide clear information about the health benefits of testing. Our analysis also suggests that to improve levels of effectiveness characterizing the current field, it may be useful to elicit commitment, and action plans, relating to how to implement testing intentions.

**Statement of contribution:**

***What is already known on this subject?***

- Interventions are urgently needed to increase HIV testing among men who have sex with men (MSM) and enable increased access to effective treatment for HIV infection.
- There is some evidence of the effectiveness of mass media interventions in increasing HIV testing among MSM.
- Nothing is known about the active components of existing mass media interventions targeting HIV testing.

***What does this study add?***

- It describes the available literature concerning evaluated mass media interventions to increase HIV testing.
- It shows few interventions report any explicit theoretical basis although many interventions share common components, including coherently connected causal mechanisms and behaviour change techniques to moderate them.
- As a minimum, future interventions should improve knowledge about testing; use well‐branded and trusted sources that endorse testing; and provide clear information about the health benefits of testing. Our analysis also tentatively suggests it may be useful to elicit commitment and planning of how to implement testing intentions.

## Background

Despite efforts to increase access to HIV testing services, it is estimated that worldwide around half of HIV‐positive people remain undiagnosed (Conserve *et al*., 2017) and Public Health England estimated that 13% of HIV‐positive men who have sex with men (MSM) were undiagnosed in 2016 (Brown *et al*., 2017). Undiagnosed infection is the primary source of onwards transmission (Skarbinski *et al*., 2015). The costs of HIV infection to society are difficult to assess, but estimated mean lifetime costs of treating one person are £360,800 with costs reducing to £179,000 if generic drugs replaced patented drugs (Nakagawa *et al*., 2012). Recent estimates suggest there are about 4,200 MSM who are currently living with undiagnosed HIV infection in the United Kingdom (Nash *et al*., 2018).

HIV testing can be thought of as a gateway to a wider range of prevention approaches (Flowers, Estcourt, Sonnenberg, Burns, 2017). Within the United Kingdom, annual HIV testing is recommended for all MSM under existing guidelines, with more frequent testing (e.g., three monthly) recommended for men reporting two or more unprotected anal intercourse partners within the last year. Despite these recommendations, McDaid *et al*. (2016) report that when combining analyses from Scotland and England using both bar‐based and online samples, appropriate levels of HIV testing are not being met; for example, annual testing was reported by only half of these UK MSM (McDaid *et al*., 2016). New behaviour change interventions are urgently needed to increase levels of appropriate HIV testing in order to capitalize on the growing range of HIV prevention approaches such as pre‐exposure prophylaxis – PrEP (Flowers, McDaid, Knussen, 2013; Flowers *et al*., 2017b; Witzel, Weatherburn, Rodger, Bourne, & Burns, 2017).

It is against this backdrop that the current study reports on one element of a wider intervention development study. Here, we report on work commissioned by a large NHS health board to conduct a review of the available literature concerning behaviour change interventions to increase HIV testing delivered through the mass media. This review work was complemented by further analyses and co‐design projects engaging community members. Cochrane review‐level evidence suggests the short‐term effectiveness and the cost‐effectiveness of mass media interventions to increase testing (Wei *et al*., 2011). Yet beyond this focus on effectiveness, there is a need to understand in more detail the active ingredients of such interventions in order to inform the development of future behaviour change interventions to increase appropriate HIV testing. A rich set of analytic tools can assist with the task of understanding the active ingredients of behaviour change interventions. Approaches such as the theory coding scheme, TCS (Michie & Prestwich, 2010), the Theoretical Domains Framework, TDF (TDFv2) (Atkins *et al*., 2017), and the Behaviour Change Techniques Taxonomy version 1 (BCTTv1) (Michie *et al*., 2013; Presseau *et al*., 2015) all share epistemological assumptions concerning the goal and value of developing cumulative knowledge to inform future interventions. These approaches, when used retrospectively within a systematic review, all seek to focus on what can be usefully learned from interventions that have been tested in the past. As such, their strengths lie in their systematic approach to specifying the shared elements of intervention content.

Many publicly funded real‐world interventions are not designed with evaluation in mind, many are not adequately theorized in relation to how their content seeks to address intervention outcomes. In parallel where there is an evaluative framework, it is often plagued by weak research designs and the use of heterogeneous outcomes. These issues can reduce the potential of analytic tools such as the TDF and the BCTT and make the rigorous development of cumulative knowledge problematic. Within this paper, we explore what can usefully be learned from the application of these approaches within one field characterized by weak evaluative designs and heterogeneous outcomes.

The analysis presented here examines the content of evaluated HIV testing interventions in relation to (1) the role of explicit (i.e., TCS) and implicit theory (i.e., TDF) within interventions (in other words, the causal, or explanatory, mechanisms addressed by the interventions); and (2) the number and details of BCTs present within these interventions (in other words, intervention elements which moderate causal, or explanatory, mechanisms); (3) in addition, working within the constraints of the available studies and their diverse designs, we tentatively explore links between theoretical domains and BCTs across the interventions and their relationship to effectiveness.

## Methods

### The review

Details of the systematic review are reported in McDaid *et al*. (2019); however, we have included a summary of the procedure undertaken for the review to provide a background to the current analysis.

### Search strategy

Five electronic databases (CINAHL, Embase, Medline, PsycInfo, and Web of Science) were searched for studies published since 2009, using detailed search strategies and standard MESH terms for HIV, MSM, and mass media interventions (for details of an example search strategy see McDaid *et al*., 2019). In addition to database searches, reference lists of included articles were searched manually. A maximum of three requests were made to study authors for intervention materials.

### Quality appraisal

No studies were excluded on the basis of quality. Details of the approach to quality appraisal can be found elsewhere (McDaid *et al*, 2013).

### Study selection

**Figure 1:**
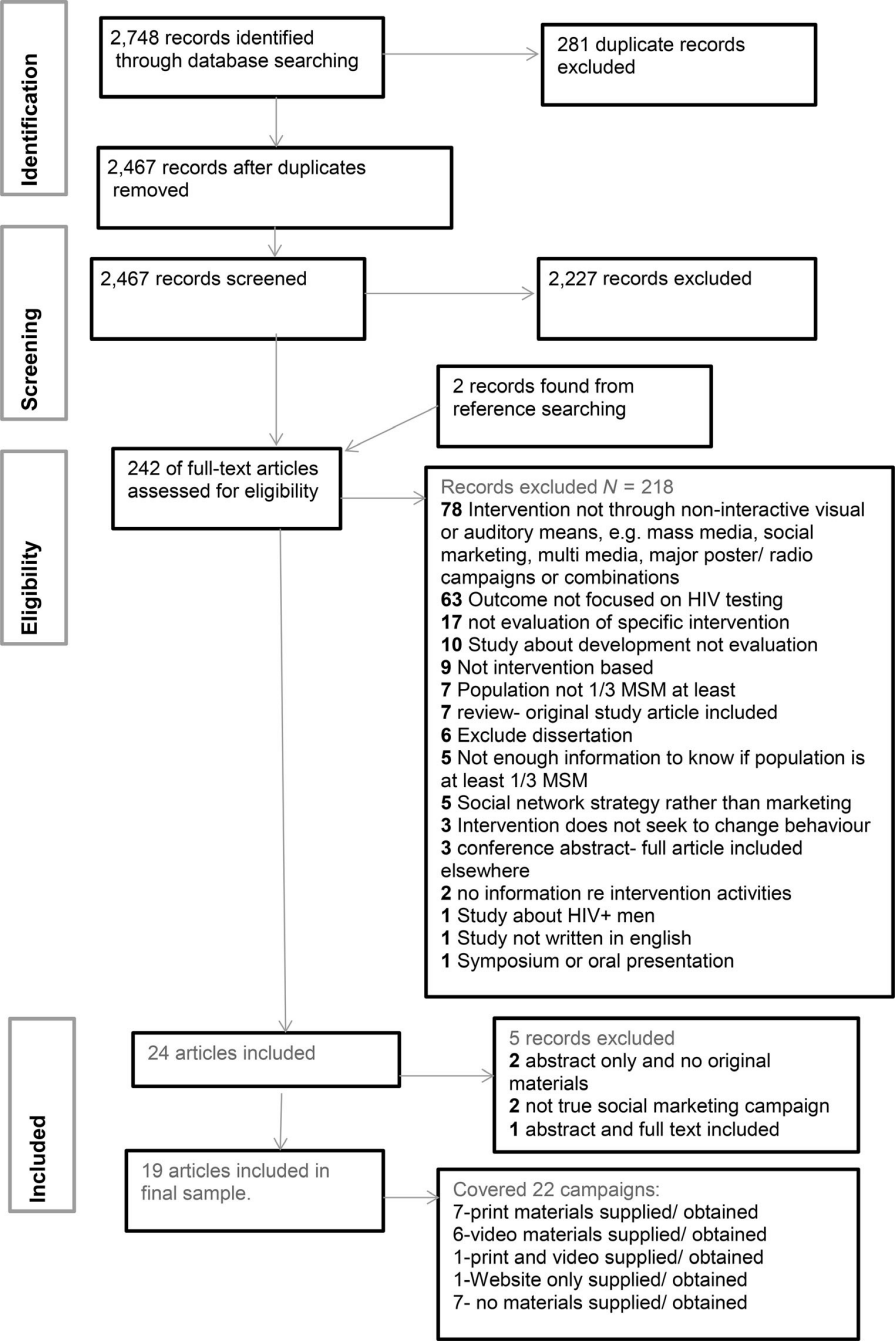
Prisma flow chart for study selection.

Studies published prior to 2010 and with intervention materials in languages other than English, Spanish, or Italian were excluded. Studies in which MSM constituted at least one‐third of the sample included interventions that sought to change behaviour through non‐interactive, visual or auditory means and included HIV testing as an outcome were included. The procedure for identifying relevant papers is shown in the PRISMA diagram in Figure 1.

### Data extraction

Structured data extraction tools were developed to capture all required information from the included studies. Where intervention materials were made available, these were used to extract data of intervention content over and above the written intervention descriptions. Where no intervention materials were available intervention descriptions were used. Data extraction was completed by one researcher with a 10% sample validated by another. Discrepancies and disagreements were resolved through consensus or through discussion with wider team members.

#### Assessing theory within the interventions

The current study uses two complementary yet distinct approaches to address theory present within the interventions: the TCS and the TDF.

In order to examine the role of explicit and formal theory within interventions, and in particular how formal theory was operationalized, the TCS was adopted (Michie & Prestwich, 2010). The current study used the first 11 items of the scheme to explore relationships between theory, target behaviours, and their role in information development and evaluation. The TCS appraises intervention content in relation to the extent it is theory driven; in other words, the way that formal theory suggests an array of theory‐driven mechanisms and hypotheses. In contrast, the TDF (Cane, O'Connor, & Michie, 2012), with its roots in implementation science, takes a more inductive approach to understanding the role of theory. As a meta‐theoretical perspective, the TDF can be used, post hoc, to code how the intervention was imagined working. In this way, it can illuminate the tacit use of theory; for example, if information provision was at the heart of what the intervention was aiming to do, there is a clear fit with the TDF domain knowledge. The TDF has a total span of 14 domains, each constituted from many more related theoretical constructs. The TDF domains provide a shared or common language in which to understand and describe the key causal mechanisms determining behaviour change across the full range of behavioural domains (be they complex behavioural domains such as the multifarious aspects of implementation or relatively simpler behavioural domains such as HIV testing). In this way, we used the TDF to conduct a retrospective assessment of the key causal mechanisms which we believed were being addressed within the interventions.

#### Behaviour change taxonomy v1

Intervention descriptions were also coded using the BCTTv1 as proposed by Michie *et al*. (2013). Intervention materials or intervention description (e.g., posters) were assessed for each of the 93 BCTs within the BCTTv1. Some elements of the intervention descriptions could be coded to multiple BCTs. In this way, we used the BCTT to code intervention elements moderating putative causal mechanisms underpinning the interventions.

#### Exploring patterns of intervention content and relative effectiveness

Given the heterogeneity of the interventions and their primary outcomes, it was not possible to conduct meta‐analytic approaches. Here, we categorize the studies to tentatively explore the patterning of theory and BCTs according to effectiveness. Effectiveness was defined by statistically significant results.

1. Intervention had a negative effect (i.e., decrease in uptake of HIV testing).
2. No evidence of a positive/negative effect.
3. Evidence of a positive effect in relation to the antecedents of HIV testing (e.g., knowledge of HIV testing increased).
4. Evidence of a positive effect on HIV testing itself.

### Analysis

TDF domains, individual and grouped BCTs were calculated across the included interventions to enable the description of ‘standard’ content, or in other words, typical, or regularly occurring content. These same elements were then mapped against the categories of effectiveness to enable the identification of unique components present only within the most effective interventions.

## Results

Nineteen articles were identified from the review, focusing on 22 separate interventions. Following repeated requests to authors, we were able to locate intervention materials for a total of 14 interventions. Table 1 presents a summary of the intervention characteristics. Interventions were all mass media interventions, incorporating combinations of photographs, video footage, and cartoon animation. They encompassed posters, radio, TV, YouTube, and other materials.

**Table 1:** Overview of included studies

Study details										Intervention effectiveness
Reference	Purpose (aim and objectives)	Design	Recruitment and data collection methods	Sample	Eligibility criteria	Exclusion criteria	Nature of intervention (s)	Control intervention	Outcome Measures	
Blas *et al*. (2010), Lima, Peru	To study the association between video‐based online interventions and proportions of HIV testing in gay‐identified and non‐gay‐identified MSM.	RCT	Online banner advertisements to redirect to study website. After consent, participant randomly assigned to condition using computer algorithm. Baseline assessment, matched emails to those attending clinic	Total = 459, non‐gay‐identified, 97 = video intervention, 90 = control (text) intervention; gay‐identified, 142 = video intervention, 130 = control (text) intervention	(1) ≥ 18 years, (2) male and report having had sex with men, (3) be a resident of Lima, Peru, (4) answer the survey from Lima, Peru (5) HIV test over 12 months ago, (5) have a valid email address and, (6) do not report being HIV positive	Excluded 937 (916 did not meet criteria, 21 did not want to participate) leaving final sample of 459. Report only results from the gay and non‐gay‐identified MSM group	Videos framed within Health Belief Model and aimed to identify strategies to overcome reasons for not testing specific to target audience.	Text used in control condition came from existing intervention to increase testing in Mexico.	Intention to get tested, actual testing	Evidence of a positive effect in relation to the antecedents of HIV testing (e.g., knowledge of HIV testing increased)
Brady *et al*. (2014), England, UK	To pilot a national, free at the point of use home HIV sampling service	Non‐comparative study	Testing rates were gathered during the intervention period	9,868 tests were requested over the pilot period and 6,230 (63.1%) were returned	Not reported	Not reported	HIV testing interventions and social media marketing were used to increase HIV testing rates, in particular those requesting self‐tests	Not applicable	Testing rates	Evidence of a positive effect on HIV testing itself
Chiasson *et al*. (2009), United States of America	To compare HIV disclosure 3 months before and after viewing intervention video	Pre‐/Post‐test study	Online banner advertisements; online self‐complete questionnaire at baseline and 3 month follow‐up.	Convenience sample: Original sample of 3,052, reduced to 442 in final sample following dropout/inclusion criteria	Limited to the 442 men who reported sex in both baseline and follow‐up interviews.	Not reported	The Morning after‐Use of 9 min dramatic video to prompt critical thinking about HIV disclosure, HIV testing, alcohol use and risky behaviours	Not applicable	Self‐reported HIV disclosure and other risk behaviours	No evidence of a positive/negative effect
Erausquin *et al*. (2009), Los Angeles County, USA	A pilot intervention to increase awareness of free testing services, provide incentives for getting test results, and improve access to treatment in Latino males.	Retrospective cohort study or cross‐sectional study	Community venues: outreach volunteers distributed cards to target population to encourage testing. Routinely gathered data from clinic with addition of information of outreach card. Data from the intervention period (August–October 2004) compared to data from two comparison periods: May–July 2004 and August–October 2003.	Convenience sample: Males testing for MSM within LAGLC's Service, Prevention, Outreach, Treatment centre in West Hollywood‐Fall 2003‐ *n* = 86, Summer 2004 *n* = 97, Fall 2004 *n* = 95	Results are limited to males who attended HIV testing within specific time frames, ≤ age 25, reporting sexual activity with a male.	Not reported	Outreach cards provided at Latino‐oriented gay club and event nights could be swapped for a movie pass at the time of testing. Information was also advertised on two Internet sites and in three gay/bisexual‐oriented magazines. Again, these included outreach cards that could be exchanged for movie passes at the time of testing.	Not applicable	Testing rates of those attending clinic	Evidence of a positive effect on HIV testing itself
Flowers, Knussen, McDaid, and Li (2013) and Flowers, McDaid, and Knussen (2013), Glasgow, Scotland	To understand the extent of self‐reported exposure to intervention among men frequenting venues for gay MSM. To explore whether sexual health‐related behaviours varied by degree of exposure to the intervention.	Cross‐sectional study	Men recruited from seven bars frequented by gay men and other MSM in Glasgow 10 months post‐intervention launch	Convenience sample: 1,313 men were approached and 822 participated, Final sample = 784 post‐exclusions	All men present or entering the venue were approached to complete a questionnaire	Final sample excluded men who identified themselves as HIV positive	Social marketing intervention aimed at MSM promoting use of condoms and water‐based lubricant during Anal intercourse; regular sexual health check‐ups and HIV testing at least every 6 months. Materials included posters, electronic images, and leaflets, with a intervention website. Posters and leaflets were distributed to both clinical and community (wider and gay scene) settings.	Not applicable	Self‐reported recency of HIV testing, recency of STI testing, Intention to HIV test, and correct use of lubricant	Evidence of a positive effect on HIV testing itself
Gilbert *et al*. (2013), British Columbia, Canada	To describe the impact of targeted NAAT on identification of AHI and discuss the potential of social marketing interventions to optimize detection among MSM.	Cross‐sectional study	Samples were included from six study clinics if sex recorded as male, transgendered or missing, and were ≥18 years	Convenience sample: Testing rates from six clinics	Sex recorded as male, transgendered or missing, and were ≥18 years	Not reported	(1) What Are You Waiting For – focused on raising awareness of rapid testing and NAAT (December 2009 to February 2010) (2) Hottest At The Start – focused on raising awareness of AHI and increased transmission risk in MSM in new relationships or engaging in risky sex. (June–August 2011).	Not applicable	Testing rates of those attending clinic	Evidence of a positive effect on HIV testing itself
Guy *et al*. (2009), Victoria, Australia	To measure the extent of any change in the uptake of testing for HIV and STIs during and subsequent to the intervention.	Cross‐sectional study	Three types of data: Sentinel surveillance data – five clinics referred to within intervention. Routine laboratory data – four clinics (pre‐intervention, during and post‐intervention). Behavioural survey – subset of existing national survey, mainly administered at gay scene event. Surveys for 2004, 2005, and 2006 were compared.	Convenience samples: those attending clinics (sentinel data/laboratory data), men completing Melbourne Gay Community Periodic Survey living in Victoria (numbers not explicitly stated)	Lab/Sentinel surveillance data – men attending clinic within set time frames. Behavioural survey – only information from Victorian residents was included	Not reported	‘Check‐It‐Out’ targeted MSM including specific groups (community/non‐community attached and ‘culturally and linguistically diverse’). Intervention aimed to increase HIV and STI testing, increase regular HIV and STI testing and promote general sexual health.	Not applicable	Lab/sentinel data: number of tests conducted per month. Behaviour study: changes in self‐reported testing patterns	No evidence of a positive/negative effect
Hickson *et al*. (2015), England, UK	Longitudinal survey to examine patterns of HIV testing and assess whether testing rates were associated with intervention periods.	Interrupted time series	Internet recruitment. Invite to enrol sent to those completing a previous survey and users of two gay‐dating websites. Self‐reported baseline survey followed by 13 monthly follow‐ups.	There were 3,386 enrolments, following exclusions/dropouts final sample of 2,047 participants.	Male; England resident; ≥16 years; sexually attracted to/has sex with men; valid email address	Those with existing HIV‐positive diagnosis and those with no or inconsistent HIV test results.	(1) ‘I Did It’ (December 2010‐April 2011)‐Terrence Higgins Trust (THT) intervention aimed to make MSM aware of ease and convenience of HIV testing. Used media advertisements, radio and website. (2) ‘Clever Dick/Smart Arse’ (November 2011–February 2012)‐THT intervention promoting condom use (3)’Count Me In’ – GMFA, encouraged men to commit to an action plan which included HIV testing.	Not applicable	Self‐reported HIV testing behaviour and self‐reported exposure to interventions	Evidence of a positive effect on HIV testing itself
Hilliam and Fraser (2011), Scotland, UK	To evaluate the impact on awareness of HIV, attitudes towards testing, prevention and safer sex in both MSM and Health Professionals	Cross‐sectional study	Internet recruitment. Websites contained link to online survey. Self‐reported online survey pre‐intervention (April–May 2010) and post‐intervention (October–November 2010). Post‐intervention recruitment added use of Grindr.	Convenience sample: Pre‐stage sample: 309 (MSM = 88; HP = 221) Post‐stage sample: 980 (MSM = 775, HP = 205)	Not reported	Men who have sex with women only	HIV Wake up Intervention (May 2010) – to inform MSM across Scotland about HIV and levels of transmission, the benefits of prevention and regular testing and where they can go to seek more information and advice. Resources included leaflets and posters, digital online banners and targeted web pages and other web media (e.g., emails targeted at Gaydar users). Materials displayed in ‘scene’ venues and wider community.	Not applicable	Self‐reported knowledge and understanding around HIV testing, awareness and exposure to intervention, HIV testing, and other risk behaviours	Evidence of a positive effect on HIV testing itself
Hirshfield *et al*. (2012), United States of America	To assess the feasibility and efficacy of implementing an online intervention (videos/HIV prevention webpage) versus a no‐content control.	RCT	Online banner advertisements with additional email sent to US members of one of the websites. Online self‐complete questionnaire at baseline and 60 days post baseline follow‐up. Participants randomly assigned to conditions	Convenience sample: Total = 3,092: Control = 609 Prevention webpage = 609, Dramatic video only = 625, Documentary video only = 633, Both videos = 616	(1) identify as male; (2) ≥18 years; (3) live in the United States.; (4) provide valid email; (5) report oral or anal sex with a current male partner (new or not), and oral, anal, or vaginal sex with at least one new partner (male or female) in the previous 60 days; (6) ability to read/respond in English	(1) lived outside of the United States; (2) identified as female, female‐to‐male transgender or male‐to‐female transgender. Duplicate cases were identified and excluded.	Five study conditions: (1) dramatic video; (2) documentary video; (3) both videos; (4) prevention webpage; and (5) control (i.e., received no intervention content). The Morning After‐drama (9 min) depicting three gay male friends, one of whom thinks he had unprotected sex with an HIV‐positive man whilst intoxicated and seeks advice from friends. Talking About HIV – documentary (5 min) HIV‐positive men discuss their experiences, uses footage from a feature‐length documentary, ‘Meth.’	Control received no content.	Self‐reported HIV disclosure and other risk behaviours	No evidence of a positive/negative effect
James (2015), England, UK	To evaluate effectiveness of English intervention, which promotes testing to men who have sex with men (MSM) and Africans.	Cross‐sectional study	Limited information: Data from testing centres and community surveys	Not explicitly stated	Not reported	Not reported	National HIV Testing week (4 weeks) promoted through targeted print, social media, and outdoor advertising. Stakeholders also provide expanded testing services.	Not applicable	Clinic‐based testing rates	Evidence of a positive effect on HIV testing itself
McOwan, Gilleece, Chislett, and Mandalia (2002), England, UK	To evaluate the effect of an HIV testing intervention specifically aimed at gay men in central London, UK, who were South European Origin, Black Origin or aged under 25 years old.	Cross‐sectional study	Convenience sample: MSM testing for HIV within one of three London clinics during 2000, laboratory records were located for those matching three target groups (South European origin, Black origin, ≤25 years)	Three clinics in London – 1999 = 65 (target clinic), 239 (other clinics); 2000 = 292 (target clinic), 236 (other clinics)	MSM testing for HIV at one of three target clinics during a specific time frame, specifically South European origin, Black origin, ≤25 years	Not reported	Gimmie 5 min (12 weeks): Advertisements in free paper distributed on the gay scene in London, images were chosen to reflect target groups	Not applicable	Testing rates at target clinic, UAI since last test, testing as result of an advert	Evidence of a positive effect on HIV testing itself
Pedrana *et al*. (2012), Victoria, Australia	To assess intervention impact using four key indicators: intervention awareness, HIV/STI knowledge, health‐seeking behaviour, and HIV/STI testing	Cross‐sectional study	Cross‐sectional data: Multiple recruitment methods: convenience samples, for example, gay community venues, gay community events; participants from a recent community‐based HIV prevalence study and snowballing. Completed online surveys, linked with unique code to allow matching, surveyed at regular intervals (3–6 monthly). Clinic data: routinely collected data from Victorian Primary Care Network for Sentinel Surveillance	Cross‐sectional data: Sample of 295 gay men Clinic data: data from three clinics	Men, ≥18 years, self‐identified as gay or homosexually active in the past 5 years. Men had to have been recruited between September 2008 and April 2009 and completed any of the three survey rounds.	Not reported	Drama Down under: Intervention aimed to increase access to treatment, increase awareness and knowledge, and minimize the transmission of HIV/STIs in MSM. Used print and radio advertisement, printed resources, outdoor advertisements, public events, and banner advertising on gay‐dating sites, ‘novel’ intervention resources (e.g., fridge magnets, drink holders, and underwear) and intervention‐specific events (e.g., the ‘Drama Down Underwear’ Show).	Not applicable	Self‐reported Awareness of intervention, HIV/STI knowledge, Testing in past 6 months, Health‐seeking behaviours. Clinic data‐testing rates	Evidence of a positive effect on HIV testing itself
Prati *et al*. (2016), Italy	To investigate the effect of intervention on performance of HIV/AIDS protective behaviours.	BA study	General population: computer‐assisted telephone survey, random digit dialling. Used Proportional quota sampling. Contacted again after 6 months. MSM participants – email lists and Web‐based communities. Self‐administered anonymous online survey, again contacted again after 6 months. Migrant participants – three survey sites: workplace, migrant shelter/camp, and centre for the teaching of Italian as a second language. Self‐administered anonymous paper‐and‐pencil survey and again after 6 months.	General population (*n* = 858), MSM (*n* = 109), and migrants (*n* = 211).	≥18 years. Took part in both pre‐/post‐surveys and sexually active in the previous 6 months.	Not sexually active in the previous 6 months before each interview	‘United Against AIDS’ (December 2012, 2 weeks; February–March 2013, 2 weeks) – television and radio public service announcements, print materials (e.g., posters, brochures), Web‐based advertisements, and cinema and newspaper advertisements. Emphasizing benefits and advantages of safer sex behaviour and getting an HIV test.	Not applicable	Self‐reported exposure to the intervention, recent (in the previous 6 months) HIV risk behaviours and lifetime HIV testing	No evidence of a positive/negative effect
Solorio *et al*. (2016), Seattle, USA	To assess intervention feasibility and identify processes that worked and those that did not.	Interrupted time series	Convenience sample: recruited from various sites, including community events, the Internet, STD clinics, entertainment venues, and Latino newspapers and referral of peers to study. Survey every 3 months, starting with 3 months before intervention (baseline interview), 3 months into, intervention and 2 months post‐intervention. Self‐reported questionnaires	Pre‐intervention assessment – 50, mid‐intervention assessment – 44, follow‐up post‐intervention – 41	(1) Self‐report Latino heritage; (2) speak Spanish; (3) biological male; (4) report sex with men in past 12 months; (5) 18–30; (6) negative HIV serostatus (if known).	Not reported	Tu Amigo Pepe: Spanish‐language radio PSAs, a Web site, social media outreach, a mobile phone reminder system, print materials, posters in stores frequented by Latinos, and a free hotline	Not applicable	Self‐reported HIV testing rates, intention, experiential attitude, instrumental attitude, self‐efficacy, and norms towards HIV testing	Evidence of a positive effect on HIV testing itself
Tang *et al*. (2016), China	To compare the effectiveness of a crowdsourced intervention versus a health marketing intervention to promote first‐time HIV testing among men who have sex with men (MSM) and transgender individuals in China	RCT	Online banner advertisement recruitment. Individuals were screened for eligibility, enrolled, and completed the survey then randomly assigned to either watch the crowdsourced video or the health marketing video. Follow‐up text message 3 weeks after survey completion asking about HIV test uptake and test result.	Total = 721 crowdsourced intervention = 352; health marketing intervention = 369	Born biologically male, having had anal sex with a man at least once, ≥16 years, never tested for HIV, provide valid mobile number.	Duplicated mobile numbers were excluded	The 1‐min video depicted two Chinese men embarking on a relationship and testing for HIV together. The 1‐min health marketing video used a cartoon storyline to provide HIV education and promoting HIV testing.	Not applicable	Self‐reported first‐time HIV testing	Evidence of a positive effect on HIV testing itself
Thackeray *et al*. (2011), USA	Provided illustrative example of the use of Social marketing theory in two case study interventions	Case study/illustrative example	Two case studies; illustrative example using social marketing theory on HIV testing intervention	Two examples	Not reported	Not reported	One on mental health, second ‘You Know Different’ – large‐scale intervention focused on increasing HIV testing among African American youth.	Not applicable	HIV testing rates	Evidence of a positive effect on HIV testing itself
West, Okecha, and Forbes (2015), England, UK	To review advertising strategies used and numbers of clients who requested POCT during NHTW.	Non‐comparative study	Grindr advertisements within 5 miles of clinics contained link to website including a video demonstrating POCT. Electronic records of those attending for POCT and activity data from software clinic	43 asymptomatic attendees	Not reported	Not reported	Grindr users within 5 miles, received link to website with POCT video, Poster interventions were also in place at the time	Not applicable	Clinic‐based testing rates and number of visits to website.	Evidence of a positive effect on HIV testing itself
Wilkinson *et al*. (2016) Victoria, Australia	To explore the effectiveness of DDU to increase HIV, syphilis, gonorrhoea, and chlamydia testing among MSM.	Cross‐sectional study	Survey data: Surveyed annually between September 2008 and August 2014. Recruitment sites varied over time, included gay venues and community events, gay sporting clubs, gay online dating sites, social media, and snowballing. Surveillance Data: The Victorian Primary Care Network for Sentinel Surveillance (VPCNSS) gathered during specific periods	1228 MSM (survey 4: *n* = 389, survey 5: *n* = 743, survey 6: *n* = 343, survey 7: *n* = 353, survey 8: *n* = 328). (242 included in final sample)	Males, self‐identifying as Gay/MSM, ≥18 years, completing 3+ surveys between December 2010 and August 2014.	Evaluation cohort: recruited pre‐December 2010, completed <3 surveys, self‐reported HIV positive. Surveillance data: Tests within 30 days of a previous test and those indicated for HIV post‐exposure prophylaxis.	Drama down under: aimed to improve screening rates and knowledge of HIV/STIs and to reduce HIV/STIs transmission among MSM. Intervention was focused on ‘inner metropolitan Melbourne’ and included outdoor media, digital media (e.g., banners on dating Web sites), and print gay media, supported by a range of intervention material (e.g., postcards, pamphlets, fridge magnets, and underwear).	Not applicable	Evaluation Cohort: self‐reported HIV test in the previous 12 months, number of partners, sex with casual partners, reporting condomless sex with casual partner, recall of intervention, and its message. Surveillance Data: HIV/STI monthly testing rates	No evidence of a positive/negative effect

### The role of explicit and implicit theory within the interventions

Using the TCS, our analysis found that only five of the included studies explicitly mentioned the use of theory, either in the development or in the delivery of the intervention (see Table 2).

**Table 2:** Theoretical Domains Framework constructs, behaviour change technique (BCT) groupings, and individual BCTs identified from intervention descriptions

	Explicit theoretical basis	Theoretical domains identified from intervention descriptions	Agreed groups of BCTs within intervention description	Agreed individual BCTs within intervention description
Blas *et al*. (2010), Lima, Peru	Health Belief Model	Knowledge	3. Social Support	3.1 Social Support (unspecified)
				3.3 Social Support (emotional)
		Social/Professional role and identity	4. Shaping Knowledge	4.1 Instruction on how to perform behaviour
		Beliefs about consequences	5. Natural consequences	5.1 Information about health consequence
				5.6 Information about emotional consequences
		Environmental context and resources	6. Comparison of behaviour	6.1 Demonstration of the behaviour
				6.2 Social comparison
		Social influences	9. Comparison of outcomes	9.1 Credible source
		Emotions	11. Regulation	11.2 Reduce negative emotions
			12. Antecedents	12.2 Restructuring the social environment
Brady *et al*. (2014), England, UK	Not reported	Beliefs about consequences	4. Shaping Knowledge	4.1 Instruction on how to perform behaviour
			5. Natural consequences	5.1 Information about health consequences
		Intentions	6. Comparison of behaviour	6.2 Social comparison
		Social influences	9. Comparison of outcomes	9.1 Credible source
			12. Antecedents	12.2 Restructuring the social environment
				12.5 Adding objects to the environment
Chiasson *et al*. (2009) United States of America	Developmental, social, and cognitive constructivist learning theories and strategies	Knowledge	1. Goals and planning	1.2 Problem solving
		Social/Professional role and identity	5. Natural consequences	5.1 Information about health consequences
				5.3 Information about social and environmental consequences
		Beliefs about consequences	9. Comparison of outcomes	9.2 Pros and cons
				9.3 Comparative imagining of future outcomes
		Social influences	16. Covert learning	16.3 Vicarious consequences
		Emotions		
Erausquin *et al*. (2009) Los Angeles County, USA	Not reported	Knowledge	4. Shaping Knowledge	4.1 Instruction on how to perform behaviour
		Social/Professional role and identity	7. Associations	7.1 Prompts and cues
		Environmental context and resources	9. Comparison of outcomes	9.1 Credible source
		Social influences	10. Reward and Threat	10.1 Material incentive (behaviour)
				10.2 Material reward (behaviour)
				10.8 Incentive (outcome)
				10.10 Reward (outcome)
Flowers, Knussen, et al. (2013) and Flowers, McDaid, et al.(2013), Glasgow, Scotland	Not reported	Knowledge	4. Shaping Knowledge	4.1 Instruction on how to perform behaviour
		Social/Professional role and identity	5. Natural consequences	5.1 Information about health consequences
		Beliefs about consequences	7. Associations	7.1 Prompts and cues
		Environmental context and resources	9. Comparison of outcomes	9.1 Credible source
			12. Antecedents	12.5 Adding objects to the environment
Gilbert *et al*. (2013) British Columbia, Canada	Not reported		4. Shaping Knowledge	4.1 Instruction on how to perform behaviour
			9. Comparison of outcomes	7.1 Prompts and cues
			7. Associations	9.1 Credible Source
		Knowledge	12. Antecedents	12.5 Adding objects to the environment
		Social/Professional role and identity		
		Beliefs about consequences		
		Beliefs about capabilities		
		Environmental context and resources		
		Social influences		
Guy *et al*. (2009), Victoria, Australia	Not reported	Knowledge	3. Social Support	3.1 Social Support (unspecified)
		Social/Professional role and identity	4. Shaping Knowledge	4.1 Instruction on how to perform behaviour
		Beliefs about consequences	5. Natural consequences	5.1 Information about health consequences
		Environmental context and resources	9. Comparison of outcomes	9.1 Credible source
			12. Antecedents	12.5 Adding objects to the environment
Hickson *et al*. (2015), England, UK	Not reported	Knowledge	1. Goals and planning	1.4 Action planning
		Beliefs about capabilities	4. Shaping Knowledge	4.1 Instruction on how to perform behaviour
		Intentions	9. Comparison of outcomes	9.1 Credible source
		Goals		
		Behavioural regulation		
Hilliam and Fraser (2011), Scotland, UK	Not reported	Knowledge	4. Shaping Knowledge	4.1 Instruction on how to perform behaviour
		Beliefs about consequences	5. Natural consequences	5.1 Information about health consequences
		Memory, attention, and decision processes	9. Comparison of outcomes	9.1 Credible source
		Environmental context and resources		
Hirshfield *et al*. (2012), United States of America	Social learning theory, situated cognition, and developmental learning theory	Knowledge	1. Goals and planning	1.2 Problem solving
		Social/Professional role and identity	3. Social Support	3.3 Social support (emotional)
		Memory, attention, and decision processes	4. Shaping Knowledge	4.1 Instruction on how to perform behaviour
		Social influences	5. Natural consequences	5.1 Information about health consequences
				5.2 Salience of consequences
				5.3 Information about social and environmental consequences
		Emotions	6. Comparison of behaviour	6.1 Demonstration of the behaviour
				6.2 Social comparison
			9. Comparison of outcomes	9.1 Credible source
				9.2 Pros and cons
			13. Identity	13.1 Identification of self as role model
			16. Covert learning	16.3 Vicarious consequences
James (2015), England, UK	Not reported	Knowledge	4. Shaping Knowledge	4.1 Instruction on how to perform behaviour
		Social/Professional role and identity	5. Natural consequences	5.1 Information about health consequences
		Beliefs about capabilities	6. Comparison of behaviour	6.2 Social comparison
		Beliefs about consequences	9. Comparison of outcomes	9.1 Credible source
		Environmental context and resources		
McOwan *et al*. (2002), England, UK	Not reported	Knowledge	3. Social Support	3.1 Social Support (unspecified)
				3.3 Social Support (emotional)
		Social/Professional role and identity	4. Shaping Knowledge	4.1 Instruction on how to perform behaviour
		Beliefs about consequences	5. Natural consequences	5.1 Information about health consequences
				5.3 Information about social and environmental consequences
				5.6 Information about emotional consequences
		Environmental context and resources	6. Comparison of behaviour	6.2 Social comparison
		Social influences	9. Comparison of outcomes	9.1 Credible source
				9.2 Pros and cons
				9.3 Comparative imagining of future outcomes
			12. Antecedents	12.5 Adding objects to the environment
Pedrana *et al*. (2012), Victoria, Australia	Not reported	Knowledge	4. Shaping Knowledge	4.1 Instruction on how to perform behaviour
		Social/Professional role and identity	5. Natural consequences	5.1 Information about health consequences
		Beliefs about capabilities	7. Associations	7.1 Prompts and cues
		Environmental context and resources	9. Comparison of outcomes	9.1 Credible source
				9.3 Comparative imagining of future outcomes
		Social influences	12. Antecedents	12.5 Adding objects to the environment
		Emotions	13. Identity	13.2 Framing/reframing
Prati *et al*. (2016), Italy	Prospect Theory	Knowledge	3. Social Support	3.1 Social support (unspecified)
				3.2 Social support (practical)
	In‐group identity theory	Social/Professional role and identity	4. Shaping Knowledge	4.1 Instruction on how to perform behaviour
	Social identity theory	Beliefs about consequences	5. Natural consequences	5.1 Information about health consequences
				5.3 Information about social and environmental consequences
		Social influences	6. Comparison of behaviour	6.2 Social comparison
		Emotions	9. Comparison of outcomes	9.1 Credible source
			13. Identity	13.2 Framing/reframing
			16. Covert learning	16.3 Vicarious consequences
Solorio *et al*. (2016), Seattle, USA	Integrated behavioural model	Knowledge	4. Shaping Knowledge	4.1 Instruction on how to perform behaviour
		Social/Professional role and identity	5. Natural consequences	5.3 Information about social and emotional consequences
		Beliefs about consequences	9. Comparison of outcomes	9.1 Credible source
				9.2 Pros and cons
		Environmental context and resources	13. Identity	13.1 Identification of self as role model
				13.2 Framing/reframing
		Social influences	16. Covert learning	16.3 Vicarious consequences
		Emotions		
Tang *et al*. (2016), China	Not reported	Knowledge	1. Goals and planning	1.9 Commitment
		Social/Professional role and identity	3. Social Support	3.2 Social support (practical)
		Beliefs about consequences	4. Shaping Knowledge	4.1 Instruction on how to perform behaviour
		Social influences	5. Natural consequences	5.1 Information about health consequences
				5.3 Information about social and environmental consequences
				5.6 Information about emotional consequences
		Emotions	6. Comparison of behaviour	6.1 Demonstration of the behaviour
				6.3 information about others’ approval
			8. Repetition and Substitution	8.2 Behaviour substitution
			9. Comparison of outcomes	9.1 Credible source
			12. Antecedents	12.2 Restructuring the social environment
			16. Covert learning	16.3 Vicarious consequences
Thackeray, Keller, Messenger, Lee Dellinger (2011), USA	Not reported	Social/Professional role and identity	4. Shaping Knowledge	4.1 Instruction on how to perform behaviour
		Beliefs about consequences	5. Natural consequences	5.1 Information about health consequences
		Environmental context and resources	9. Comparison of outcomes	9.1 Credible source
		Social influences	12. Antecedents	12.5 Adding objects to the environment
			13. Identity	13.2 Framing/reframing
West *et al*. (2015), England, UK	Not reported	Knowledge	4. Shaping Knowledge	4.1 Instruction on how to perform behaviour
		Social/Professional role and identity	6. Comparison of behaviour	6.1 Demonstration of the behaviour
		Beliefs about consequences	7. Associations	7.1 Prompts and cues
		Environmental context and resources	9. Comparison of outcomes	9.1 Credible source
Wilkinson *et al*. (2016), Victoria, Australia	Not reported	Knowledge	4. Shaping Knowledge	4.1 Instruction on how to perform behaviour
			5. Natural consequences	5.1 Information about health consequences
			9. Comparison of outcomes	9.1 Credible source

Blas *et al*. explicitly discussed use of the Health Belief Model (Rosenstock, 1974). Solorio *et al*. (2016) was the only study to identify and use a single theory, the Integrated Behavioural Model, which drew upon a range of other theories. In contrast, Chiasson, Shaw, Humberstone, Hirshfield, & Hartel (2009), Hirshfield *et al*. (2012), and Prati, Mazzoni, Cicognani, Albanesi, & Zani (2016) drew upon multiple theoretical perspectives simultaneously, linking intervention content to a range of formal but generic theoretical perspectives, including social identity theory (Tajfel, 2010; Turner, Hogg, Oakes, Reicher, & Wetherell, 1987), social and cognitive learning theory, social learning theory, situated cognition, and constructivist learning theory (Bandura, 1986; Brown, Collins, & Duguid, 1989; Driscoll, 2000; Lave & Wenger, 1991; Schank & Berman, 2002; Shuchat Shaw & Chiasson, 2007).

In relation to the TDF and the implicit use of theory, the interventions included in this study worked to increase HIV testing through focusing upon causal mechanisms such as the provision of ‘knowledge’ (*n* = 18), utilizing *social roles and identities* (*n* = 15), sexual or ethnic identities, and by focusing recipients’ ‘beliefs about the consequences’ of their HIV testing‐related behaviours (*n* = 14). Intervention content also sought to change behaviour through changing the ‘environment context and resources’ often by using particular spaces to prime behaviour change (*n* = 13). Finally, intervention content also worked through ‘social influence’ (*n* = 12) using mechanisms such as social identification or social norms around testing to cue behaviour change.

### Behaviour change techniques

Our analysis examined BCTs identified within interventions in terms of both BCT groupings (*n* = 16) and individual BCTs (*n* = 93).

In relation to BCT groups, within the included studies, 13 distinct groups of BCTs were employed, with the three most common groupings being ‘Comparison of outcomes’, ‘Natural consequences’ and ‘Shaping knowledge’ (see Table 2). In terms of individual BCTs, our findings identified 30 distinct BCTs were used across the interventions. We have grouped these BCTs into three main categories relating to frequency of use within the interventions: those that are *very* common in most interventions (*n* = 10–30), those that are *fairly* common (*n* = 5–10), and those that are *rarely* used (*n* < 4).

Our findings indicate that three ‘very common’ BCTs that form the backbone of most of the included interventions and could therefore be considered ‘standard’ content: ‘Instructions on how to perform behaviour’ (*n* = 19 studies), ‘Credible source’ (*n* = 19), and ‘Information about health consequences’ (*n* = 15).

The group of ‘Fairly common’ BCTs were often linked to each other and to those very commonly used (see above). They could be considered as reinforcing this ‘standard content’. For example, ‘Information about social and environmental consequences’ (*n* = 7) clearly relates to the more prevalent technique of ‘Information about health consequences’ and encourages the recipient to consider the wider consequences of HIV testing for self, others, and the wider community beyond the consequences associated with health. Other fairly common BCTs which could be linked to contributing to the decision‐making process regarding adopting, or not, the desired behaviour (HIV testing) were also identified within the contributing interventions. ‘Social comparison’ (*n* = 7), ‘Pros and cons’ (*n* = 5), and ‘Vicarious consequences’ (*n* = 5) encourage the recipient to consider their behaviours either in relation to perceived or observed consequences to self and/or others and task recipients with a decisional balance or reckoning of the reasons to test for HIV. Interventions employing video narratives were most likely to adopt the technique of ‘Vicarious consequences’ by depicting the consequences of testing and/or risk behaviour within a social setting. Similarly, the implementation of the BCT ‘Social comparison’ (*n* = 6) was often visual and sometimes nuanced, relying on the recipients to identify with the images or characters stories within the interventions. Other fairly common techniques adopted, related to the provision of resources, either through ‘social support (unspecified)’ (*n* = 5) and related technique ‘social support (emotional)’ (*n* = 4) or through ‘adding objects to the environment’ (*n* = 7). For example, several studies identified the use of approaches such as credit card‐shaped leaflets that could be kept within the wallet. This technique linked to the use of ‘Prompts and Cues’ (*n* = 6), where materials were often situated in locations such as venues where testing could take place in order to prompt or cue testing.

In terms of the ‘rarely used’ BCTs, our findings suggest that interventions tended to employ BCTs that could be described as relatively passive and designed for delivery through one‐off implementation.

We also identified key absences within the intervention content in that we found that BCTs relating to the groups ‘feedback and monitoring’ and ‘goals and planning’ appear to be under‐used or absent within the included interventions. This potentially reflects the constraints of modes of delivery used within these mass media interventions.

### Exploring patterns of intervention content and relative effectiveness

Elsewhere, we detail the results of our analysis of the effectiveness of these interventions (McDaid *et al*, 2013). The majority of studies (*n* = 12/19) reported results that were indicative of positive change in relation to HIV testing, whilst only five of the studies included indicated that the intervention had no effect.

In relation to explicit use of theory, and the degree to which we can say these interventions were theory driven, given the small numbers of studies explicitly discussing theory, we were unable to examine relative patterns of effectiveness in any meaningful way by the results of the TCS analysis.

In relation to implicit use of theory, or the key causal mechanisms that underpinned the included interventions, the TDF domains ‘beliefs about capabilities’, ‘intentions’, ‘goals’, and ‘behavioural regulation’ were only observed in interventions that showed evidence of a positive effect on HIV testing. Many of the more widely used TDF domains were present across at least two of the four categories of intervention effectiveness.

Across all categories of relative effectiveness, most of the groupings of BCTs (12/16) were observed. Interesting distributions were also observed in terms of patterning of individual BCTs across the interventions when they were grouped in relation to our four categories of intervention effectiveness. Single occurrences of ‘Problem Solving’ and ‘Salience of consequences’ were only observed in interventions showing no evidence of a positive, or negative, effect. In contrast, individual BCTs that featured within interventions showing evidence of a positive effect on HIV testing included one‐off instances of ‘Action planning’, ‘Commitment’, ‘Information about others approval’, ‘Material incentive (behaviour)’, ‘Information Material reward (behaviour)’, ‘Incentive (outcome)’, ‘Reward (outcome)’, ‘Reducing negative emotions’. Equally, ‘Information about emotional consequences’ and ‘Restructuring the social environment’ were observed within the effective interventions more often (three times each).

In relation to the BCTs, on average interventions reporting no effect were coded with a larger number of BCTs than those reporting a positive effect. However, some of these ineffective interventions were delivered by video, and therefore, we would suggest that the number of BCTs employed may be a reflection of intervention complexity and interactions with form of delivery rather than illuminating effectiveness *per se*.

## Discussion

The current study has, for the first time, described the typical active content of previously evaluated international interventions designed to change HIV testing behaviour among MSM through the use of mass media. Moreover, within the constraints of heterogeneous study designs and diverse study outcomes, we have also tentatively explored patterns of effectiveness related to this active intervention content. These results represent a step forwards in directing future HIV testing intervention design for this population as they lever a level of hitherto under‐specified detail regarding potentially useful intervention content. Our analysis specifies both the causal mechanisms, and the BCTs, used in many of these, largely effective, interventions. Our analysis also tentatively suggests the potential of further BCTs that may be uniquely associated with effectiveness. In this way, the analysis progresses the field but also demands further work for intervention development.

Across the contributing studies, our use of the TCS showed that interventions in this field are rarely theory driven; it was difficult to see coherent connections between these explicit formal theories and the active intervention content we identified. In other words, there is a disconnect between formal theory when it is mentioned, and the actual active elements of the interventions identified. It may be that formal theory is conceptualized in relation to the rather generic behaviour change goals of the interventions, rather than assisting with specifying particular content, which is tailored to the known barriers and facilitators of HIV testing behaviour. Alternatively, it may be that theory is added *post hoc* in relation to the discussion of the findings.

Our use of the TDF highlighted a strong sense of the key causal mechanisms which these interventions address. Whilst the links between the theoretical constructs reflected within formal theory and intervention content could be described as opaque, the TDF domains which these interventions address are logical, plausible, and work well with the medium of mass media. The interventions identified within the review focus on *knowledge* provision; speak directly to *social roles and identities* primarily in relation to gay men and gay cultures; have a clear focus upon targeting and attempting to change intervention recipients’ *beliefs about the consequences* of HIV testing; address the *environmental and contextual* determinants of testing within the gay milieu; and capitalize on *social influence* particularly in relation to the social dynamics between gay men. In this way, these interventions collectively have a logical and coherent approach to addressing plausible causal mechanisms which may change HIV testing behaviour among MSM.

In relation to the BCT groups we identified, the three most common groupings were ‘Comparison of outcomes’, ‘Natural consequences’, and ‘Shaping knowledge’. ‘Comparison of outcomes’ and ‘Natural consequences’ are similar in that both BCT groups encourage the recipient to consider the consequences of behaviour change; the first elicits a weighing up of options (e.g., pros and cons of testing or not), whilst the second focuses the participant to think through the consequences of testing, or not. ‘Shaping knowledge’ focuses on BCTs related to providing information and instruction about how to perform the behaviour (e.g., where to access HIV testing). Again, there is a clear sense of these groups of BCTs being logical and plausible intervention elements that may be useful in changing testing behaviour. Our analysis of individual BCTs showed very commonly used BCTs included; ‘Instructions on how to perform behaviour’, ‘Credible source’ and ‘Information about health consequences’. Again, these aspects of active intervention content are logical and work well with the mass media format of intervention delivery.

Looking across the intervention content we identified, there is a clear sense of congruence between the tacit theory identified using the TDF (i.e., the causal mechanisms the interventions addressed) and the BCTs we could identify from the best available intervention descriptions and materials (i.e., the intervention content intended to change behaviour). In this way, there was good evidence of the underlying logic, or what could be described as a tacit theory of change, that underpinned many of these interventions. This logic tracked an arc connecting the likely determinants of behaviour (i.e., TDF domains) with congruent intervention elements that were logically tailored to them (i.e., BCT groupings and individual BCTs). For example, the theoretical domain of *knowledge* clearly relates to the BCT grouping ‘Shaping knowledge’ and concomitant BCTs ‘information about health consequences’ and ‘instructions on how to perform a behaviour’. Equally, the TDF domain ‘beliefs about consequences’ relates well to both BCT groupings, ‘Comparison of outcomes’ and ‘Natural Consequences’. In this way, the findings can be interpreted to suggest that BCTs that provide factual information from a trusted source and that also demonstrate support for desired behaviour change (i.e., HIV testing) and foster the intervention recipients understanding of the consequences of behaviour may be particularly common building blocks within mass media interventions to increase HIV testing. Our findings suggest that the underlying logic of these approaches is concerned with embedded BCTs that enable testing decisions based on credible information about the relative benefits of enactable behaviour change. In relation to the pragmatic aims of the project and our funders desire to develop an evidence‐based mass media HIV testing intervention, our analysis of these published evaluations highlights the backbone of these largely effective interventions as being concerned, in the main, with a series of coherent and logically connected intervention elements. We believe these represent foundational elements for future interventions likely to yield similar degrees of effectiveness as those that contributed to the current review.

Our analysis of the patterning of BCTs across our categories of relative effectiveness is also interesting although limited given the heterogeneity of contributing studies and their outcome measures. Although it is not based on statistical analysis, it does suggest very specific ideas to take forwards into future intervention development work. It suggests TDF domains such as ‘beliefs about capabilities’, ‘intentions’, ‘goals’, and ‘behavioural regulation’ may be useful in improving intervention effectiveness beyond levels of effectiveness anticipated by the standard content outlined above. It also suggests that BCTs such as ‘Action planning’ or ‘Commitment’ may be worthy of further detailed exploration. These BCTs are interesting as they include a temporal element inviting intervention recipients to consider their behaviour in the future. It is worth noting that the use of these BCTs may be constrained by the mode of delivery within the mass media. It may be useful to consider how using the digital media may enable more interactive and sequential engagement with mass media interventions. Such sequential engagement may help with the formation of plans to implement intentions to test. Other BCTs worthy of further exploration include those which address the social and emotional aspects of HIV testing, these include ‘Information about others approval’, ‘Reducing negative emotions’, ‘Information about emotional consequences’, and ‘Restructuring the social environment’. These BCTs are interesting as they acknowledge the non‐health‐related aspects of HIV testing and the immediate social context of HIV echoing many of the key local barriers to HIV testing (Flowers *et al*.,^2013^).

### Implications for practice, policy, and further research

This study represents the first attempt to describe and interpret the active content of mass media interventions intended to increase HIV testing in gay men and other MSM. It provides hitherto unarticulated detail regarding the standard content of such interventions and makes granular suggestions of potentially useful future intervention content. It is useful as it provides a benchmark, albeit a snapshot, of the active content of recent mass media interventions to change HIV testing behaviour among MSM.

### Limitations

Our analysis is limited by the legacy effects of the contributing studies. The included papers are mainly from the developed world and do not address the full range of social and cultural contexts inhabited by MSM who may benefit from increased HIV testing. We caution against the straightforward generalization of the findings reported here to MSM populations who do not receive the benefits of the social equality they deserve (e.g., in countries in which homosexuality is criminalized such as Jamaica, Saudi Arabia, or Uganda).

Our analysis is also limited by the range of materials describing the intervention content that were made available to the team. We systematically requested such materials three times from the authors of the included studies; 14 sets of intervention descriptions were eventually received. Intervention materials tended to provide richer accounts of the intervention content than that provided within published descriptions of interventions included within the primary evaluation papers. As such although we were consistent in using the best available intervention descriptions, it is important to note that poor reporting of intervention content within primary papers cautions against any simplistic interpretation of the findings reported here.

Furthermore, it is important to acknowledge that our synthesis and assessment of the patterns of intervention content and their relative effectiveness is not statistical. We have tried to glean useful information from the available evidence, but this does not relate to the specification of effect sizes or statistical insights into relative contribution of specific intervention elements to overall intervention effectiveness.

A further key limitation relates to our lack of appraisal of the cultural, technological, and temporal contexts within which these interventions were delivered. Whilst this was beyond the scope of the current paper, it begs questions to how effectiveness, and the role of specific active intervention content, may well relate to complex interactions between the intervention and the context it was delivered in. These limitations relate to wider weaknesses of the TDF and BCW approaches and stem from their tendency to deracinate interventions, and intervention content, from the key contexts in which they were implemented. Similarly, whilst the high degree of granularity associated with these approaches enables the specification of highly specific accounts of intervention content, it does not lend itself to understanding how diverse intervention components can work either synergistically, or inharmoniously. This particular point is relevant to considering how to interpret the specific BCT content of interventions, where interdependencies between BCTs may well be important in understanding effectiveness.

The team have already addressed some of these limitations with additional analyses (Flowers et al., in preparation), in which the findings reported within the current paper were synthesized with other diverse sources of evidence. These additional sources of evidence included: (1) a TDF and Behaviour change wheel (Michie, Atkins, & West, 2014) analysis of the perspectives from local gay men and other MSM about the immediate context and culture, in order to specify culturally appropriate intervention elements tailored to local men, (2) a synthesis, and subsequent analysis using the TDF and behaviour change wheel (Michie *et al*., 2014) of the international literature concerning barriers and facilitators to HIV testing to specify theory‐informed, and evidence‐based, potentially useful intervention content to complement the suggestions identified within the current study.

### Conclusions

Our approach, detailing active intervention content in relation to TDF domains and BCTS, has identified key intervention elements that are likely to be useful to develop future mass media interventions to promote appropriate HIV testing. ‘Standard’ content was identified in most interventions: improving knowledge about testing, well branded and trusted sources endorsing the message and clear information about the health benefits of testing. However, it is also important to consider the use of BCTs that may boost or reinforce this ‘standard content’, for example, extending recipients understanding of the consequences of testing to include sexual partners and communities or eliciting commitment and planning of how to implement testing intentions. Further research that examines these issues within the local context and population is recommended to complement the results described here.

## Funding

This study was funded by NHS Greater Glasgow and Clyde. PF, LMcD, and JR are funded by the UK Medical Research Council (MRC) and Scottish Government Chief Scientist Office (CSO) at the MRC/CSO Social & Public Health Sciences Unit, University of Glasgow (MC_UU_12017/11, SPHSU11 and MC_UU_12017/12, SPHSU12). NB was funded by the UK Medical Research Council (MRC) and Scottish Government Chief Scientist Office (CSO) at the MRC/CSO Social & Public Health Sciences Unit, University of Glasgow (MC_UU_12017/11, SPHSU11).

## Disclaimer

Nicky Coia contributed to the research process, up to and including drafting of the paper. The funding bodies had no other role in the preparation or submission of the manuscript, and the views expressed are those of the authors alone.

## Authors’ contributions

LMcD and PF co‐designed and sought funding for the project. LMcD led the overall study, with PF overseeing data extraction and analysis pertinent to this paper, which were conducted by JR and NB. All authors were actively involved with each step of the research processes up to and including drafting of the paper. All authors approved the final version.
